# Effect of an Active Video Game Intervention Combined With Multicomponent Exercise for Cardiorespiratory Fitness in Children With Overweight and Obesity: Randomized Controlled Trial

**DOI:** 10.2196/33782

**Published:** 2022-05-24

**Authors:** Cristina Comeras-Chueca, Lorena Villalba-Heredia, Jose Luis Perez-Lasierra, Gabriel Lozano-Berges, Angel Matute-Llorente, German Vicente-Rodriguez, Jose Antonio Casajus, Alex Gonzalez-Aguero

**Affiliations:** 1 Department of Physiatry and Nursing Faculty of Health Science University of Zaragoza Zaragoza Spain; 2 GENUD (Growth, Exercise, Nutrition and Development) Research Group Zaragoza Spain; 3 EXERNET Red de Investigación en Ejercicio Físico y Salud para Poblaciones Especiales Zaragoza Spain; 4 Department of Physiatry and Nursing Faculty of Health and Sport Science Universidad de Zaragoza Zaragoza Spain; 5 Instituto Agroalimentario de Aragón-IA2 Centro de Investigación y Tecnología Agroalimentaria de Aragón Universidad de Zaragoza Zaragoza Spain; 6 Centro de Investigación Biomédica en Red de Fisiopatología de la Obesidad y Nutrición Madrid Spain

**Keywords:** active videogames, VO2peak, obesity, prepuberty, cardiorespiratory, fitness, gaming, childhood, intervention

## Abstract

**Background:**

Childhood overweight and obesity have become major global health problems and are negatively related with the cardiorespiratory fitness (CRF) level in school children and adolescents. Exercise, specifically multicomponent training, is effective for CRF improvement, but the main challenge is to ensure adherence to exercise in children with overweight and obesity. Therefore, new ways of exercising that are more attractive and motivational for this population are needed and playing or training with active video games (AVGs) has been proposed as an effective alternative because they require full-body movement and therefore increase energy expenditure.

**Objective:**

The main aim of this study was to investigate the effects of an AVG intervention combined with multicomponent training on CRF at maximal and submaximal intensities in children with overweight or obesity.

**Methods:**

We recruited 28 children (13 girls and 15 boys) aged 9 to 11 years with overweight or obesity from medical centers and divided them into 2 groups, an intervention group (n=20) that participated in a 5-month supervised AVG exercise program combined with multicomponent exercise, and a control group (n=8) that continued daily activities without modification. A maximal stress test to measure CRF using a walking-graded protocol with respiratory gas exchange was performed by the participants.

**Results:**

The AVG group showed a significant decrease in heart rate and oxygen uptake for the same intensities in the submaximal stages of the maximal treadmill test, as well as a lower oxygen uptake percentage according to the individual maximal oxygen uptake, whereas the control group did not show overall changes. No change in the peak oxygen uptake (VO_2peak_) was found.

**Conclusions:**

A 5-month AVG intervention combined with multicomponent exercise had positive effects on CRF at submaximal intensity, showing a lower heart rate and oxygen uptake at the same intensities and displaying a lower oxygen uptake percentage according to the individual (VO_2peak_). Greater benefits were found in children with the highest fat percentage.

**Trial Registration:**

ClinicalTrials.gov NCT04418713; https://clinicaltrials.gov/show/NCT04418713

## Introduction

### Background

Childhood overweight and obesity have become major global health problems [1,2]. The World Health Organization even refers to obesity as a “global pandemic” [3]. The worldwide prevalence of childhood overweight and obesity remains high, but the rising trends have plateaued in many high-income countries [4]. Nevertheless, the prevalence of overweight was over 30% and that of obesity was over 10% in European children and adolescents in 2016 [5]. This high prevalence is still worrisome because childhood obesity has important health implications such as the increased risk of developing cardiovascular and cardiometabolic diseases (eg, type 2 diabetes, hypertension, or metabolic syndrome), psychosocial problems (eg, low self-esteem, low self-confidence, low self-efficacy, low motivation for physical activity, bullying, or difficulties in establishing relationships) [6,7], and an increased risk of becoming overweight or obese adults [3].

Overweight and obesity have been shown to be negatively related with cardiorespiratory fitness (CRF) levels in school children and adolescents [8,9]. The relationship between both factors has also been found in preschoolers [10] and becomes more pronounced as children grow older [11], suggesting that high CRF levels should be promoted as early as possible as a preventive measure because poor CRF is even associated with the development of cardiometabolic risk factors [12,13] and metabolic syndrome [14]. Preschool BMI is also inversely associated with fitness in adolescence [15]. It is noteworthy that a higher proportion of girls than boys have reduced aerobic capacity [16]. Among all physical fitness variables, the peak oxygen uptake (VO_2peak_) shows the strongest inverse relationship with BMI and fat mass or body fat percentage [17]. Based on the inverse relationship between CRF and body fat [18,19], it seems that the cutoff point for the negative effects of fatness depending on CRF starts above 16%-20% relative body fat content [18].

On the other hand, an inverse reciprocal relationship was observed between motor competence, and VO_2peak_ and body fatness during childhood [20,21]; therefore, improving motor competence will go hand in hand with improving CRF in the objectives of an exercise intervention.

Exercise, specifically multicomponent training, is effective for improving CRF [22,23]. However, the main challenge is to ensure adherence to exercise in children with overweight and obesity [24]. Therefore, new ways of exercising that are more attractive and motivational for this population are needed.

Playing or training with active video games (AVGs) has been proposed as an effective alternative to exercise and can be as effective as moderate exercising; AVGs are being investigated to determine their effectiveness against childhood obesity. AVGs generally require full-body movement and therefore increase energy expenditure [25,26]; nevertheless, the effects of AVG interventions on CRF are unclear due to the lack of evidence. In fact, the few studies investigating the effects of AVGs on CRF use indirect methods to assess CRF such as the shuttle run test, step test, or The Progressive Aerobic Cardiovascular Endurance Run test. This is a limitation in detecting positive effects at submaximal intensities. In addition, it should be noted that AVG interventions need to be supervised and structured to ensure their effectiveness in improving physical fitness or increasing physical activity [27,28].

### Objective

The main aim of this study was to investigate the effects of an AVG intervention combined with multicomponent training on CRF at maximal and submaximal intensities in children with overweight or obesity.

## Methods

### Ethics Approval

The ethical guidelines of the 1964 Declaration of Helsinki (revised in Fortaleza, 2013) [29] and the Declaration of Taipei [30] were followed in the conduct of this study. The protocol was reviewed and approved by the Research Ethics Committee of the Government of Aragón (certificate number 11/2018, CEICA, Spain). Written informed consent was obtained from all participants and their parents or guardians, after being informed of the nature and possible risks of the experimental procedures in the study.

### Study Overview

This randomized controlled trial (RCT) is part of a larger cross-over study (trial registration number: NCT04418713). In this RCT, participants were divided into 2 groups, an intervention group that participated in the AVG exercise program combined with multicomponent exercise, and a control group that continued daily activities without modification.

The recruitment process was carried out through pediatricians from the medical centers. Informative talks about the activity were given in medical centers, and it was the pediatricians themselves who proposed the activity to patients with overweight or obesity who could benefit from the activity. Due to the difficulty of this recruitment process, it was planned to extend the study for another year to obtain a bigger sample. Therefore, a 2:1 randomization was carried out in the first year, prioritizing the AVG intervention. SPSS (version 22.0; SPSS Inc) was used to generate the random allocation sequence and it was performed by the researchers who carried out the project. In the second moment, randomization was going to be 2:1, favoring the control group; unfortunately, it was interrupted by the COVID-19 pandemic and the final groups were not uniform. The second part of the study with a new recruitment process was also interrupted by the COVID-19 pandemic.

### Participants

The sample consisted of 28 children (13 girls and 15 boys) with overweight or obesity recruited from medical centers through their pediatricians or from the schools of Zaragoza (Spain). Participants met the following inclusion criteria: aged between 9 and 12 years, Tanner I or II (assessed through direct observation by a physician) and not having had menarche, overweight or obesity calculated by BMI and following the cutoff points of Cole and Lobstein [31], without contraindications for the practice of physical exercise, and without pathologies that worsen with physical exercise. In addition, the following were the exclusion criteria: participating in regular high-level or high-intensity extracurricular physical activities, following any special diet regime, and taking any medication that may interfere with the variables evaluated. The parents and pediatricians were informed about the development of the activity, results, and progress of the children through briefings.

### Intervention

The participants were requested to attend 3 sessions per week, lasting approximately 60 minutes each, and the intervention lasted 5 months. The sessions were composed of a regime with a 10-minute warm up, including joint mobility; dynamic flexibility; muscle activation; and core, balance, and coordination exercises. This was followed by the main part, which consisted of 45 minutes of exercise with a combination of AVG and multicomponent exercise, followed by a circuit training dynamic where the participants continuously rotated from AVG to exercises, and finally a 5-minute cooldown part to lower the heart rate (HR) and end the session with static flexibility routines. In general, the sessions consisted of 4 AVGs with an average duration of 8 minutes, and the multicomponent exercise was performed between the AVG sessions. The multicomponent exercise lasted 13 minutes on average per session, divided into 2 or 3 activities with different objectives depending on the planning. Several physical activity and sport professionals supervised the sessions.

In the main part, the AVGs included were the following: the Xbox 360 with Kinect using “Kinect Adventures” and “Kinect Sports;” the Nintendo Wii using “Wii Sports;” “Just Dance” and “Mario and Sonic at the Olympic Games;” dance mats using “Dance Revolution” and “Mario and Sonic at the Olympic Games” adapted from the Nintendo Wii to the dance mats; and the BKOOL interactive cycling simulator connected to a HUAWEI MediaPad T5 AGS2-W09 tablet. The intervention was carried out in 2 locations, the University of Zaragoza and the San Braulio public school in Zaragoza. All the AVGs were provided through funding, and each site was equipped with the AVGs necessary to develop the intervention. The sessions were different every day, following a progression in difficulty and intensity and fulfilling the objectives previously established during planning. The participants did not play all the AVGs in each session, so the number of sessions recorded for each AVG was different. The order in which the activities were carried out was different among the participants, as each participant started in an AVG and changed it after playing.

The AVGs were combined with multicomponent exercises focused on enhancing health-related physical fitness, such as CRF, muscular endurance, and muscular strength, along with coordination and balance. This intervention design combining AVGs with traditional exercise was selected due to a potentially greater energy expenditure [32]. The multicomponent exercise performed had a playful background to enhance motivation and enjoyment.

### Outcomes

#### Anthropometry

All the participants underwent anthropometric examination wearing minimal clothing. Height was measured to the nearest 1 mm with a stadiometer (SECA 225, SECA) and weight to the nearest 0.1 kg with an electronic scale (SECA 861, SECA). BMI was calculated as the weight (kg) divided by the square of the height (m^2^).

#### CRF Measurements

A walking-graded protocol was employed to assess cardiovascular fitness. The test was performed on a treadmill (Quasar Med 4.0, h/p/cosmos) with a face mask fitted. The tests were carried out in the laboratory of the GENUD (Growth, Exercise, Nutrition and Development) research group. The test was explained to the participants, who were fitted with electrodes and had their resting HR measured before starting. After fitting the safety harness, the test started at a comfortable walking pace (3.2 km/h), and the speed was increased by 0.8 km/h every 2 minutes until the participants walked quickly (5.6 km/h), which was the maximum speed reached during the test. Then the slope was increased by 4% every minute until exhaustion or up to a maximal slope of 24%. A sports medicine physician supervised the entire test and performed a preclinical examination to determine if the participant was suitable for performing the stress test. The respiratory gas exchange data were measured breath by breath using open-circuit spirometry (Oxycon Pro, Jaeger/Viasys Healthcare).

Peak values of the VO_2_ and HR were defined as the highest average values obtained for any continuous 15-second period. The metabolic cart was calibrated daily with a known gas and volume as recommended by the manufacturer.

The HR was continuously recorded using 12-lead electrocardiography (H12+, Mortara Instrument) from the beginning to the end of the stress test. The maximal HR value was the highest HR value recorded during the last stage of exercise. The blood pressure was also measured with a digital monitor (M3, HEM-72OO-E, Omron Healthcare Europe), for health and safety reasons, before the maximal effort test with the participant lying in a tilt, and during the recovery period in the standing position, both on the right arm. The cuffs were adjusted to the circumference of the tested arm, and the measurement was taken twice. The participants had to be at rest 5 min before the pretest measurement.

### Statistical Analyses

SPSS (version 22.0, SPSS Inc) was used to perform all the statistical analyses. Statistical significance was set at *P*<.05 in all tests. Data are presented as means and SDs. Kolmogorov-Smirnov tests were performed to verify the normal distribution of the variables; several variables did not show a normal distribution, and therefore nonparametric tests were performed.

The Mann-Whitney *U* test was conducted to examine differences between the AVG group and control group for descriptive characteristics and CRF parameters before and after intervention, whereas the Wilcoxon test was performed for within-group comparisons among the preintervention and postintervention measures. The biserial correlation coefficient (r) was calculated using the formula of Fritz et al [33] for nonparametric contrasts/comparisons and the following thresholds were considered: small effect (>0.1), medium effect (>0.3), and large effect (>0.5) [34].

In addition, 2 groups were created based on the baseline body fat percentage using the 50th percentage of the sample to investigate the effect of body fat on the participants' response after the AVG intervention and multicomponent exercise. Low and high CRF categories were established using the 50th percentile based on sex published by Johansson et al [35] for children with overweight and obesity. The reference 50th percentile values for the relative maximal oxygen uptake in boys and girls were 30.8 and 30.6 mL/kg/min, respectively.

The percentage of the VO_2peak_ achieved by all the participants at each stage was calculated through the objective data of their VO_2peak_ obtained in the maximal exercise test. The percentage of change from the pretest measurement to the posttest measurement was calculated for the HR, VO_2peak_, and percentage of the VO_2peak_.

## Results

### Participant Characteristics

The descriptive variables of the participants included in this study are shown in Table 1. No differences between the groups at baseline were found.

No differences between groups were observed in preintervention measurements. Age, weight, height, and lean mass significantly increased in the AVG and control groups from the pretest to the posttest (*P*<.05). The AVG group showed a decreased body fat percentage and BMI z-score (*P*<.05). For the control group, the VO_2peak_ (L/min) increased (*P*<.05).

All the participants completed at least 75% of the sessions, showing good adherence to this AVG intervention combined with multicomponent exercise.

**Table 1:** Characteristics of the subjects in active video game and control groups.

Variable		Total (N=28)	AVG^a^ group (n=20)	Control group (n=8)
**Age (years), mean (SD)**				
	Preintervention	10 (0.8)	10.2 (0.8)	9.7 (0.8)
	Postintervention	10.7 (0.8)	10.7 (0.8) *	10.5 (0.8)*
**Weight (kg)** **, mean (SD)**				
	Preintervention	53.3 (9)	55.3 (9)	48.2 (7.2)
	Postintervention	56.2 (10)	57.7 (9.7)*	52.5 (10.3)*
**Height (cm)** **, mean (SD)**				
	Preintervention	144.6 (7.7)	146 (6.9)	141.1 (8.8)
	Postintervention	148.9 (7.5)	149.6 (7.3)*	147 (8.3)*
**BMI (kg/m^2^)>, mean (SD)**				
	Preintervention	25.3 (2.8)	25.8 (3)	24.1 (1.7)
	Postintervention	25.2 (3)	25.7 (3.1)	24.1 (2.8)
**BMI z-score** **, mean (SD)**				
	Preintervention	1.95 (0.3)	1.98 (0.4)	1.89 (0.2)
	Postintervention	1.84 (0.4)	1.88 (0.42)*	1.72 (0.3)
**BMI percentile** **, mean (SD)**				
	Preintervention	96.8 (2.2)	96.8 (2.6)	96.8 (1.2)
	Postintervention	95.6 (4.1)	95.8 (4.6)	95.1 (2.5)
**Body fat percentage, mean (SD)**				
	Preintervention	40.9 (4)	41.3 (3.7)	39.8 (4.7)
	Postintervention	40.1 (4.4)	40.1 (4.5)*	39.9 (4.4)
**Lean mass (kg), mean (SD)**				
	Preintervention	29.7 (4.9)	30.6 (4.7)	27.4 (5)
	Postintervention	31.8 (5.5)	32.6 (5.1)*	29.7 (6.3)*
**VO_2peak_^b^ (mL/kg/min), mean (SD)**				
	Preintervention	32.85 (5.9)	32.75 (5.9)	33.1 (6.2)
	Postintervention	33.3 (5.3)	32.8 (5.5)	34.7 (4.6)
**VO_2peak_ (L/min), mean (SD)**				
	Preintervention	1.72 (0.29)	1.77 (0.21)	1.6 (0.42)
	Postintervention	1.85 (0.29)	1.86 (0.27)	1.81 (0.37)*

^a^AVG: active video games.

^b^VO_2peak_: peak oxygen uptake.

*Significant differences within groups between preintervention and postintervention (*P*<.05).

### Effects of the AVG Intervention Combined With Multicomponent Exercise on HR at Submaximal and Maximal Effort

The effects of the intervention using AVGs combined with multicomponent exercise on the HR for maximal and submaximal efforts are detailed in Table 2. No differences between groups were found neither before nor after the intervention for any HR variable (*P*>.05). Nevertheless, the results showed a significant decrease in the maximal HR of the AVG group (*r*=0.535) and at every submaximal stage: 3.2 km/h (*r*=0.505), 4 km/h (*r*=0.577), 4.8 km/h (*r*=0.689), 5.6 km/h (*r*=0.765), and 5.6 km/h with a slope of 4% (*r*=0.480). Lower HR values were observed for the same intensities, whereas the control group did not show any changes.

**Table 2:** Heart rates in the different submaximal stages of the maximal stress test by group (N=28).

Heart rate at various levels (bpm^a^), mean (SD)			AVG^b^ group (n=20)		Control group (n=8)
**Speed: 3.2 km/h; slope: 1%**					
	Preintervention	118.2 (12.44)		120.38 (10.31)	
	Postintervention	113.7 (10.6)*		116.38 (9.49)	
	% change	–3.45 (7.19)		–3.08 (7.21)	
**Speed: 4 km/h; slope: 1%**					
	Preintervention	127.6 (13.86)		124 (11.51)	
	Postintervention	121.75 (11.48)*		122.75 (10.22)	
	% change	–4.27 (6.34)		–0.75 (6.71)	
**Speed: 4.8 km/h; slope: 1%**					
	Preintervention	137.75 (15.42)		133.13 (13.04)	
	Postintervention	129.9 (10.97)*		130.13 (9.6)	
	% change	–5.28 (6.22)		–1.91 (6.34)	
**Speed: 5.6 km/h; slope: 1%**					
	Preintervention	152.3 (17.42)		146 (14.78)	
	Postintervention	143.40 (14.43)*		142.63 (12.98)	
	% change	–5.59 (5.4)		–2.03 (7.03)	
**Speed: 5.6 km/h; slope: 4%**					
	Preintervention	157.2 (37.76)		155.5 (16.57)	
	Postintervention	153.95 (15.74)*		152 (12.88)	
	% change	–5.48 (44.53)		–1.92 (6.05)	
**Maximum HR^c^ (bpm)**					
	Preintervention	197.1 (12.24)		191.63 (10.51)	
	Postintervention	187.2 (22.42)*		191.75 (6.94)	
	% change	–4.86 (11.1)		0.2 (3.75)	

^a^bpm: beats per minute.

^b^AVG: active video game.

^c^HR: heart rate.

*Significant differences within the group between preintervention and postintervention (*P*<.05).

### Effects of the AVG Intervention Combined With Multicomponent Exercise on Submaximal and Maximal Effort Oxygen Uptake and Length of the Maximal Treadmill Test

**Figure 1:**
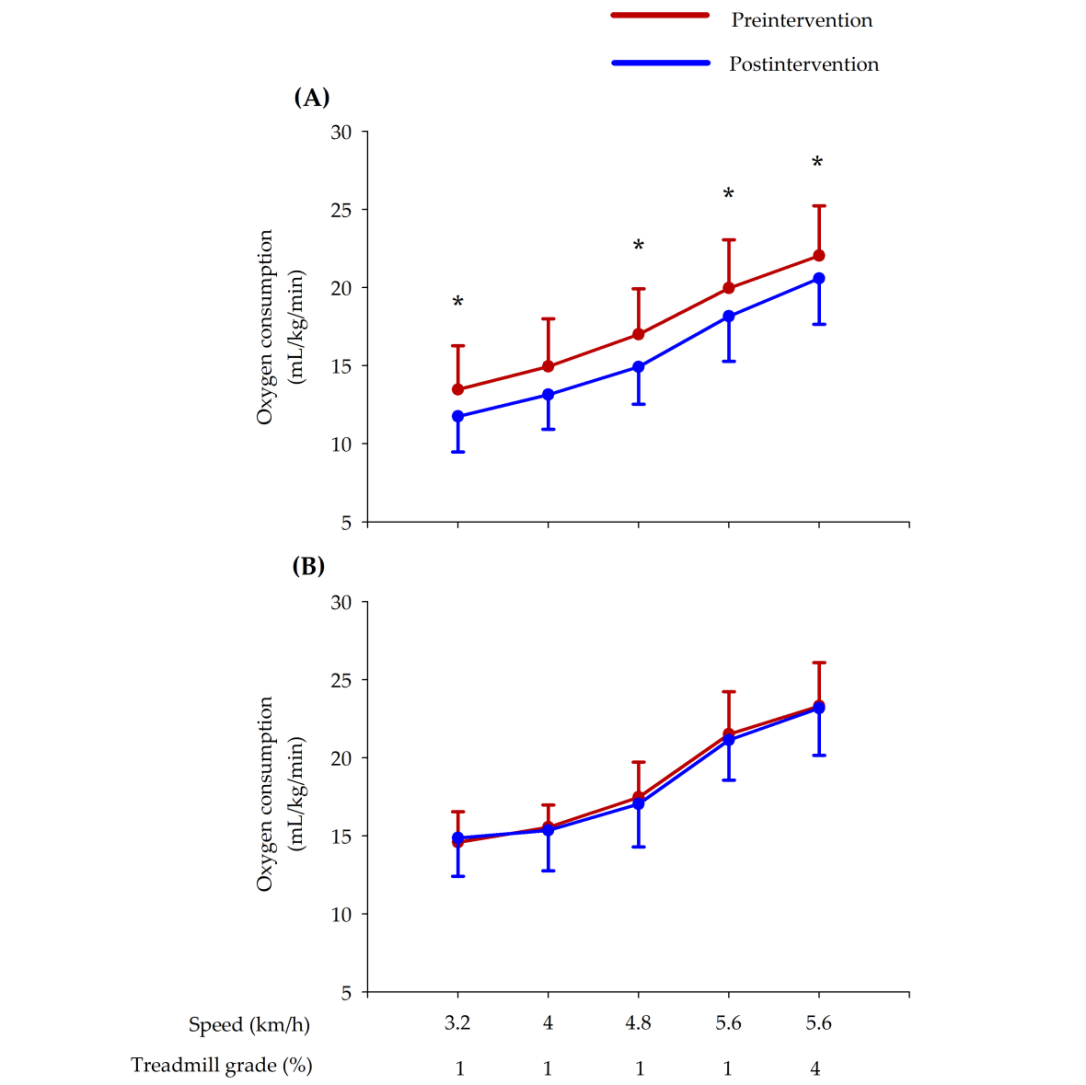
Changes in VO_2_ during the different stages of the maximal stress test observed for the (A) active video game group and (B) control group.

The effects of the intervention using AVGs combined with multicomponent exercise on VO_2_ for the maximal and submaximal efforts are detailed in Table 3. No pretest differences were found between groups (*P*>.05). After the AVG intervention combined with multicomponent exercise, a decrease in the submaximal VO_2_ was found, but no effects were reported for the VO_2peak_. As shown in Figure 1, lower VO_2_ values occur for the same intensities after the intervention at every submaximal stage of the test: at 3.2 km/h (*r*=0.518), 4 km/h (*r*=0.434), 4.8 km/h (*r*=0.593), 5.6 km/h (*r*=0.535), and 5.6 km/h with a slope of 4% (*r*=0.551). The control group did not show any change in the VO_2_ parameters. In addition, significant differences between groups were found in the submaximal VO_2_ after the AVG intervention, with significantly lower VO_2_ in the AVG group at 3.2 km/h and 5.6 km/h. The duration in minutes of the maximal treadmill test significantly increased in the AVG and control groups, with no differences observed.

**Table 3:** Oxygen uptake in the different submaximal stages of the maximal stress test by group (N=28).

Variable, mean (SD)			AVG^a^ group (n=20)	Control group (n=8)
**VO_2_^b^ (mL/kg/min); speed: 3.2 km/h; slope: 1%**				
	Preintervention	13.47 (2.81)		14.59 (1.96)
	Postintervention	11.76 (2.26)*		14.86 (2.46)^**^
	% change	–8.73 (27.85)		2.8 (17.3)
**VO_2_ (mL/kg/min); speed: 4 km/h; slope: 1%**				
	Preintervention	14.95 (3.05)		15.55 (1.43)
	Postintervention	13.15 (2.22)		15.34 (2.58)
	% change	–8.73 (23.65)		–1.13 (15.43)
**VO_2_ (mL/kg/min);speed: 4.8 km/h; slope: 1%**				
	Preintervention	17.02 (2.9)		17.48 (2.25)
	Postintervention	14.92 (2.39)*		17.03 (2.73)
	% change	–10.41 (17.92)		–2.06 (13.55)
**VO_2_ (mL/kg/min); speed: 5.6 km/h; slope: 1%**				
	Preintervention	19.97 (3.09)		21.50 (2.72)
	Postintervention	18.16 (2.88)*		21.13 (2.56)^**^
	% change	–7.49 (18.56)		–0.47 (16.13)
**VO_2_ (mL/kg/min); speed: 5.6 km/h; slope: 4%**				
	Preintervention	22.05 (3.18)		23.30 (2.78)
	Postintervention	20.59 (2.93)*		23.18 (3.03)
	% change	–4.54 (21.04)		–0.04 (11.43)
**VO_2peak_ (mL/kg/min)**				
	Preintervention	32.75 (5.9)		33.11 (6.18)
	Postintervention	32.82 (5.54)		34.68 (4.65)
	% change	1.23 (13.08)		6.6 (15.36)
**Length of the maximal treadmill test (min)**				
	Preintervention	11.38 (0.94)		11.16 (0.95)
	Postintervention	12.37 (1.20)*		12.42 (0.58)*
	% change	8.76 (5.58)		11.72 (7.13)
**Length of the effort phase of the maximal treadmill test (min)**				
	Preintervention	3.39 (0.92)		3.25 (0.98)
	Postintervention	4.38 (1.16)*		4.42 (0.58)*
	% change	36.34 (44.57)		44.4 (38.15)

^a^AVG: active video game.

^b^VO_2_: oxygen uptake.

* Significant differences within groups between preintervention and postintervention (*P*<.05).

^**^Significant differences between AVG and control groups (*P*<.05).

In addition, the VO_2_ percentage was calculated for each participant according to the VO_2_ peak obtained from the maximal treadmill test. Results reported no differences between groups before the AVG intervention. Based on the VO_2_ values in mL/kg/min, the AVG group showed significant decreases in the VO_2_ percentage at all submaximal stages of the maximal treadmill test: 3.2 km/h (*r*=0.609), 4 km/h (*r*=0.551), 4.8 km/h (*r*=0.693), 5.6 km/h (*r*=0.576), and 5.6 km/h with a slope of 4% (*r*=0.476). On the other hand, the control group showed decreases in the VO_2_ at only 4.8 km/h (*r*=0.792). Significant differences in the VO_2_ were found between the AVG and control groups after the AVG intervention at 3.2 km/h.

### Effects of the AVG Intervention Combined With Multicomponent Exercise on Submaximal and Maximal Effort Oxygen Uptake Percentage by Groups According to Body Fat Percentage Baseline in the AVG Group

As explained before, the sample was divided into 2 groups according to the 50th percentile of the body fat percentage for the AVG group. The results showed that the participants with a higher body fat percentage showed a significantly decreased HR and VO_2_ at the same intensities after the intervention. Specifically, lower HR values were found for the participants in the AVG group with higher body fat percentages at 3.2 km/h (*r*=0.737), 4 km/h (*r*=0.662), 4.8 km/h (*r*=0.751), 5.6 km/h (*r*=0.886), and 5.6 km/h with a slope of 4% (*r*=0.864); furthermore, lower HR values were found for the AVG group participants with lower body fat percentages at only 4.8 km/h (*r*=0.454). On the other hand, lower VO_2_ values were found for the AVG group participants with higher body fat percentages in the submaximal stages of the maximal treadmill test at 4 km/h (*r*=0.617), 4.8 km/h (*r*=0.697), and 5.6 km/h (*r*=0.885); in contrast, the AVG group participants with lower body fat percentages did not show any changes. However, the influence of the body fat percentage on the VO_2_ percentage determined from the individual VO_2peak_ of the maximal treadmill test was low, showing that the AVG group participants with higher body fat percentages displayed significant decreases in the VO_2_ percentage during the submaximal stage at 4.8 km/h (*r*=0.590) and 5.6 km/h (*r*=0.751). However, the AVG group participants with lower body fat percentages displayed significant decreases in the VO_2_ percentage during the submaximal stages at 3.2 km/h (*r*=0.691) and 4.8 km/h (*r*=0.810). No significant differences between the AVG group participants with higher and lower body fat percentages were found in the baseline and posttest.

## Discussion

### Principal Findings

The aim of this study was to investigate the effects of an AVG intervention combined with multicomponent training on CRF at maximal and submaximal effort levels in children with overweight and obesity. The main finding of this study was the significant decrease in the HR and VO_2_ shown by the AVG group for the same intensities at the submaximal stages of the maximal treadmill test, along with a lower VO_2_ percentage according to the individual maximal oxygen uptake. As a reference, the control group did not show overall changes in the HR, VO_2_, or VO_2_ percentage in the submaximal stages of the maximal treadmill test, except for a significant decrease in the VO_2_ percentage at 4.8 km/h. However, no changes in the relative or absolute VO_2peak_ values (in mL/kg/min and L/min) were found for the AVG group, although an increment in the absolute VO_2peak_ value was observed for the control group. This might be due to the weight gain in the control group, mainly fat accumulation. Although we did not expect improvements in the VO_2peak_, the adequate intensity level required for achieving the desired improvements at the maximum intensities was not reached. This may be explained by the nature of the intervention limiting the intensity to that produced by the AVGs and the inability of the participants to reach such demanding intensities due to excess body fat. However, one of the objectives of the intervention was to improve quality of life by enabling these children to better cope with daily activities, which are often submaximal efforts.

Regarding the CRF endurance level of the participants, mean VO_2peak_ values of 32.75 (SD 5.90) mL/kg/min for the AVG group and 33.11 (SD 6.18) mL/kg/min for the control group were obtained. The children with overweight and obesity who participated in this study showed low CRF levels with an increased risk of health problems due to this low level of CRF in 75% of the participants, according to the cutoff points proposed by Ruiz et al [36]. Moreover, 67.9% of the participants are above the 50th percentile according to Johansson et al [35], which means that the participants in this study had a higher CRF compared to the mean of the normative data of children with overweight or obesity.

In relation to maximal exercise testing, it is difficult for children, especially those with overweight or obesity, to meet all the maximal criteria and demonstrate a plateau in their maximal oxygen uptake [37]. To determine if the exercise test was maximal and the VO_2peak_ data were valid, the percentage of the theoretical maximal HR reached at the end of the test and the respiratory exchange ratio≥1.15 [38] were used. Only 4 of the 28 participants in the preintervention maximal exercise test and 3 of the 28 participants in the postintervention maximal exercise test did not reach 90% of their theoretical maximum HR at the end of the maximal stress test, but they achieved a respiratory exchange ratio very close to (1.13 and 1.14), equal to, or higher than 1.15. Therefore, it can be strongly believed that the tests were maximal.

Thus far, only 7 studies have investigated the effects of AVG interventions on the CRF of children with overweight or obesity, and the results are unclear. The first study that reported the effects of AVGs on the CRF of adolescents with overweight and obesity was that by Adamo et al [39] and the results showed a significant training effect over time with two different interventions: AVG cycling and stationary cycling with music interventions, with sessions of 60 minutes twice per week for 10 weeks. Both interventions produced significant improvements in the peak HR, peak workload, or the time to exhaustion, along with significant reductions in the body fat percentage; however, no significant differences were found between the exercise groups. With this same intervention, Goldfield et al [40] observed that the psychological benefits of these aerobic exercises were related to improved aerobic fitness. These positive effects are in line with our results. Although they show that the positive effects of AVG cycling on CRF are comparable to those of the stationary cycling with music intervention, adherence to this stationary cycling with music intervention was greater. Maddison et al [41] found decreases in body fat percentage with no significant increases in the CRF for the AVG group, measured by the 20-m shuttle test. However, this positive effect of AVGs on body composition in children with overweight or obesity is most likely mediated through improved aerobic fitness [42]. This relationship between body fat percentage and CRF supports the results of this study in which participants with higher body fat percentage had greater improvements in CRF. The interpretation of this result could be that participants with higher body fat percentage have a greater facility for decreasing body fat, which is associated with an increase in CRF. Maloney et al [43] observed no improvements in CRF of the AVG and control groups, assessed by a 3-minute step test after playing Dance Dance Revolution for 12 weeks. This discrepancy may be due to the method used to measure CRF; the participants did not reach adequate intensities and the study did not report the number of sessions per week and the duration of these sessions, which could also explain the lack of positive effects. Christison et al [44] showed that the number of shuttle runs did not change after a 6-month AVG intervention, with 2 sessions per week using several devices. This study has several limitations that could explain the lack of positive effects of the AVG intervention such as the small sample, the difference in the number of participants between the intervention and control groups, the short length of the intervention whose duration was 10 weeks, and the method for measuring the CRF for which the 20-m shuttle run test was used. The most recent study was conducted by Bonney et al [45], who investigated the effect of Wii Fit, in comparison with a task-oriented functional training, on the performance in the shuttle run test and positive effects on CRF in both groups. However, no differences between the AVG and control groups performing the task-oriented functional training were found after a 14-week intervention conducted once a week with each session lasting for 45 minutes. Furthermore, 2 noncontrolled trials studied the effects of AVG on CRF in children with overweight and obesity [46,47]; the limitation of these trials was the lack of a control group, which means that the results should be interpreted with caution. Calcaterra et al [47] observed an improvement in CRF (3.8 mL/kg/min, *P*<.001) measured by a walking test on a treadmill reaching 85% of the maximal HR after a 12-week intervention using interactive video games. Huang et al [46] showed no effects of AVGs using Nintendo Wii and Xbox Kinect on CRF after 16 sessions, probably due to the inclusion of only 14 participants and the short length of the intervention. A systematic review performed by Zeng and Gao [48] included only 1 RCT [41], which reported positive effects of an AVG intervention in comparison with an exercise group, but these results were unclear due to the inclusion of only 1 study. Given the lack of studies on the usefulness of AVGs to improve CRF, more quality research investigating AVGs as tools to improve CRF is needed. In addition, systematic reviews on AVGs that include CRF are needed, given that CRF is one of the key components of health-related physical fitness and is closely related to quality of life and health.

On the other hand, when the AVG group was divided in 2 groups according to body fat percentage, the results showed that the participants with higher body fat percentage showed significantly decreased HR and VO_2_ values at the same intensities after the intervention, which translates into improved efficiency during submaximal efforts. As stated above, participants with higher body fat percentages had a greater range of improvement, and therefore, it is easier for them to achieve improvements, showing significant decreases in the HR and VO_2_ at the same submaximal intensities after the AVG intervention combined with multicomponent exercise. However, smaller improvements were observed in participants with lower body fat percentages. These results are supported by previous studies considering the effect of AVGs on CRF, although there are none that determine CRF with breath-by-breath measurements, indirect calorimetry, and the maximal stress test along with gas exchange measurements. As previously stated, there are several studies that support the use of AVGs to improve CRF in children with overweight or obesity. None of these studies compare the outcomes in children based on body fat, and they do not differentiate between overweight and obesity. However, the studies investigating the use of AVGs to improve CRF in children with a healthy weight indicate low effectiveness; among the 6 controlled trials, 3 studies [49-51] report positive effects and the other 3 studies [52-54] report no effects, contrary to the studies involving children with overweight or obesity, most of which found significant improvements in CRF.

### Strengths and Limitations

Some limitations must be considered in this study. The number of participants was low, especially in the control group. This could make it more difficult to identify the effects of the AVG intervention and achieve enough statistical power. Another important limitation is the unequal number of participants in the AVG and control groups because the control group could not be completely formed owing the COVID-19 pandemic, which interrupted research activities worldwide.

However, some strengths can be highlighted. The intervention was supervised and structured, with a duration (5 months) and frequency (3 sessions per week and 60 minutes per session) similar to or higher than that of any other previous AVG intervention reporting benefits for this population. Another important aspect is the combination of AVG and multicomponent training focused on CRF, muscular strength, agility, and coordination. In addition, the wide variety of AVGs used should be highlighted. All these devices offer opportunities and possibilities to significantly increase energy expenditure in children with overweight or obesity [26]. Finally, the main strength was the maximal stress test conducted to measure CRF using a walking-graded protocol with respiratory gas exchange measurements that allowed HR and VO_2_ values to be recorded at submaximal intensities.

### Conclusions

A 5-month intervention of AVGs combined with multicomponent exercise had positive effects on CRF at submaximal intensity, showing lower HR and VO_2_ values at the same intensities and lower VO_2_ percentages according to the individual VO_2peak_ values. The body fat percentage and the BMI z-score were also reduced after the AVG intervention. In addition, greater improvements were found in children with the highest fat percentage.

Future research could focus on the design and implementation of AVG interventions with higher intensities than those used in this study to produce improvements in CRF at submaximal and maximal effort levels, as this type of intervention appears effective for children with overweight or obesity.

## Appendix

Multimedia Appendix 1 CONSORT-EHEALTH checklist (V 1.6.1).

## Abbreviations

AVG: active video game
CRF: cardiorespiratory fitness
HR: heart rate
RCT: randomized controlled trial
